# Impact of Disruption and Drying Conditions on Physicochemical, Functional and Antioxidant Properties of Powdered Ingredients Obtained from Brassica Vegetable By-Products

**DOI:** 10.3390/foods11223663

**Published:** 2022-11-16

**Authors:** Claudia Bas-Bellver, Cristina Barrera, Noelia Betoret, Lucía Seguí

**Affiliations:** Instituto Universitario de Ingeniería de Alimentos para el Desarrollo, Universitat Politècnica de València, 46022 Valencia, Spain

**Keywords:** brassica, glucosinolates, isothiocyanates, agro-industrial by-products, antioxidant properties, functional powders

## Abstract

Reintroducing waste products into the food chain, thus contributing to circular economy, is a key goal towards sustainable food systems. Fruit and vegetable processing generates large amounts of residual organic matter, rich in bioactive compounds. In Brassicaceae, glucosinolates are present as secondary metabolites involved in the biotic stress response. They are hydrolysed by the enzyme myrosinase when plant tissue is damaged, releasing new products (isothiocyanates) of great interest to human health. In this work, the process for obtaining powdered products from broccoli and white cabbage by-products, to be used as food ingredients, was developed. Residues produced during primary processing of these vegetables were transformed into powders by a process consisting of disruption (chopping or grinding), drying (hot-air drying at 50, 60 or 70 °C, or freeze drying) and final milling. The impact of processing on powders’ physicochemical and functional properties was assessed in terms of their physicochemical, technological and antioxidant properties. The matrix response to drying conditions (drying kinetics), as well as the isothiocyanate (sulforaphane) content of the powders obtained were also evaluated. The different combinations applied produced powdered products, the properties of which were determined by the techniques and conditions used. Freeze drying better preserved the characteristics of the raw materials; nevertheless, antioxidant characteristics were favoured by air drying at higher temperatures and by applying a lower intensity of disruption prior to drying. Sulforaphane was identified in all samples, although processing implied a reduction in this bioactive compound. The results of the present work suggest Brassica residues may be transformed into powdered ingredients that might be used to provide additional nutritional value while contributing to sustainable development.

## 1. Introduction

The Brassicaceae (or Cruciferae) family comprises about 3200 species, including cruciferous vegetables of commercial interest such as cabbage, cauliflower, broccoli and mustards, as well as oilseed and condiment crops and ornamental plants, among others [1,2]. A characteristic of cruciferous plants is the synthesis of the secondary metabolites named glucosinolates [3,4]. Glucosinolates (GSLs) consist of a generic chemical structure formed by the thiohydroximate-O-sulfonate group linked to glucose and a variable aglycone side chain (R-chain) derived from one of eight amino acids [4,5,6]. These bioactive chemicals play an important role in biotic stress resistance, specifically in the defence against herbivores and microorganisms [6,7]. GSLs role in plant defence is mainly due to their interaction with the enzyme myrosinase [8]. Once plant tissue damage occurs because of chewing, heating, grinding, digestion, or insect or herbivore attack [5,7], myrosinase hydrolyse GSLs producing a variety of bioactive and/or toxic compounds [5] such as isothiocyanates, thiocyanates, nitriles, goitrin and epithionitriles, depending on the precursor molecule, reaction conditions (temperature and pH) or the presence of certain cofactors [4,9]. Interest regarding the human health-promoting effects of GSLs and their hydrolyzation products such as isothiocyanates is increasing, which particularly applies to sulforaphane as one of the most abundant in brassicas [10]. Several in vitro and in vivo studies have reported the protective function of these compounds against diabetes or cardiovascular diseases [5]; moreover, the chemopreventive activity, anti-inflammatory effect and epigenetic mechanism’s regulation of brassica-derived phytochemicals in humans has also been investigated [4,6,11,12,13]. These health-promoting effects present an opportunity for the development of anti-inflammatory and chemopreventive functional foods, dietary supplements and drugs [11].

A large amount of fruit and vegetable wastes are generated along the whole food chain. In the very first stages of processing, these are due to discards because of high commercialization standards, as well as to the removal of non-desired or damaged parts. In primary production, losses in vegetables, fruits, tubers and roots vary from 10 to 30% of the production volume [14]. Brassica varieties such as broccoli or white cabbage generate large quantities of residual organic matter that are not consumed or commercialized but are still rich in bioactive compounds. These varieties are widely produced in Europe: in 2020, more than 4 million tonnes of cabbage and 2 million tonnes of cauliflowers and broccoli were produced, with Spain producing 7% and 34%, respectively [15].

Towards the development of a circular economy system and the reduction in food loss and wastes, there is the need for developing new production systems and products that minimize waste and reintroduce residues into the food chain. These approaches contribute to the development of more sustainable food systems [16] focusing on the four priorities: nutrition and health, climate and sustainability, circularity and resource use, and innovation and communities. 

Fruit and vegetable by-products and discards, essentially outer layers and extremities, contain large amounts of bioactive compounds such as dietary fibers, antioxidant compounds and others [17], with beneficial effects on human health [18,19], and a great potential for sustainable and functional food product development. These materials in raw form are susceptible to microbial spoilage, so their transformation into powdered ingredients is an interesting alternative. Transformation of these wastes into powders implies a rather simple processing resulting in versatile products, rich in bioactive compounds, with increased physicochemical and microbiological stability [17,18]. These powdered ingredients offer wide applicability in the food industry, improving the nutritional characteristics of foodstuffs, as food preservatives and additives (colourings, flavourings, etc.), and might be incorporated into meat and bakery products [17,18,20] or added to prepared foods such as instant soups [21] or infant formulations [20]. Furthermore, and particularly with brassica powders, the products could have potential uses in agriculture as a natural resource for pest control and disease prevention in crops due to their glucosinolates and isothiocyanates content [22,23].

Powder manufacturing requires the use of several stages (cleaning, grinding, drying, powdering), which in turn determine the properties of the products obtained. Thus, an important step before production is the assessment of the impact of processing parameters on the powders’ properties. The intensity of disruption can affect the properties of the products as, for instance, excessive damage to plant tissue may negatively affect the content of antioxidant compounds [24]. Moreover, factors such as the size of the particles generated, the contact surface with air or the internal structure of the matrix generated are decisive for the dehydration process and therefore for the characteristics of the powders obtained [25]. Likewise, the effect of dehydration depends on the type of product and its structure, as well as on preprocessing stages and drying conditions, such as air temperature [26,27]. The advantages and disadvantages of different dehydration techniques commonly applied to develop functional ingredients and foods have been previously discussed [28]. In this review, air drying and freeze drying are proposed as appropriate drying methods for the integral valorisation of vegetable wastes and discards.

In this context, the present work was developed under a project in collaboration with the agricultural cooperative Agrícola Villena Coop. V., aiming to transform these brassica wastes into powdered food ingredients. Therefore, the purpose of the present work was to develop the process for obtaining powdered products from broccoli (*Brassica oleracea* var. *italica*) and white cabbage (*Brassica oleracea* var. *capitata*) residues (stems and outer leaves, respectively), and their subsequent physicochemical and functional characterization in order to evaluate the impact of processing on the powder’s properties and their applications.

## 2. Materials and Methods

### 2.1. Raw Material and Powder Manufacturing

White cabbage (*Brassica oleracea* var. *capitata*) and broccoli (*Brassica oleracea* var. *italica*) residues were used in this work. White cabbage wastes, corresponding to the outer leaves generated in the manufacturing lines for the commercialization of fresh cabbage, were provided by the cooperative Agrícola Villena Coop. V. (Alicante, Spain). Broccoli was purchased from a local supermarket in Valencia (Spain) and residue (stems) was separated manually in the laboratory with a knife. Fresh vegetable discards were disinfected with a 1% (*v*/*v*) sodium hypochlorite solution in water. 

The first step in obtaining the powders was to disrupt the tissue with a Thermomix^®^ TM6 food processor (Vorwerk, Madrid, Spain) into pieces of ≤10 mm diameter, called hereinafter chopped (C), or into ground (G) pieces of ≤5 mm diameter. Disruption conditions were set according to preliminary tests, as in Bas-Bellver et al. (2020) [29]. Chopping was carried out at 5000 rpm for 5 s and grinding at 10,000 rpm for 10 s. Wastes were then dehydrated by hot-air drying (HAD) or freeze drying (FD). HAD was carried out in a convective CLW 750 TOP+ transverse flow tray dryer (Pol-Eko-Aparatura SPJ, Katowice, Poland) with an air velocity of 2 m/s and drying temperature of 50, 60 or 70 °C. Drying conditions were decided based on preliminary experiences. Ground or chopped residues were distributed on the dryer trays (~200 g of residue/tray) in 10 mm thick layers. Convective drying was conducted until water activity (a_w_) measurements on the samples taken from the trays were reduced to 0.3, to guarantee stability of the final product [26]. FD consisted of deep-freezing ground samples at −40 °C in a CVN-40/105 freezer (Matek, Barcelona, Spain) and further freeze drying in a LyoQuest-55 laboratory freeze drier (Telstar, Terrasa, Spain) at 0.1 mbar and −45 °C (condenser temperature). Once dehydrated, the product obtained, either air-dried or freeze-dried, was ground to obtain a fine powder (10,000 rpm at 30 s intervals for a total 2 min milling) in a Thermomix^®^ TM6 food processor (Vorwerk, Madrid, Spain). The powders obtained were packed into glass jars in a light-free environment. 

Hereinafter, powdered products are identified according to the type of residue: white cabbage (WC) or broccoli (B); the pretreatment applied: grinding (G) or chopping (C); and the drying method used: HAD50, HAD60 and HAD70 for HAD at 50, 60 and 70 °C, or FD for freeze drying.

### 2.2. Matrix Behaviour during Air Drying: Drying and Drying Rate Curves

Both the weight and the water activity of samples were registered at different times throughout the drying process as explained elsewhere [29]. This procedure allowed the drying curves of the Brassica vegetable wastes to be obtained at 50, 60 and 70 °C, as well as to register the time required to reach a water activity value (a_w_) lower than 0.3. For white cabbage outer leaves, this took 16 h at 50 °C, 14 h at 60 °C and 12 h at 70 °C, and for broccoli stems, 15 h at 50 °C, 10 h at 60 °C and 8 h at 70 °C. 

The drying curves (moisture on dry basis vs. time) and the drying rate curves (drying rate vs. moisture on dry basis) were obtained by stating dry matter balances between the final conditions of drying (M_f_: sample weight at the end of drying; x_wf_: sample moisture content at the end of drying) and each measuring time along the drying process (M_t_: sample weight at time t; x_wt_: sample moisture content at time t) following Equation (1). A dry matter balance states that the amount of dry matter remains constant along the drying process and the only mass transfer taking place corresponds to the water being removed from the product.

M_t_·(1 − x_wt_) = M_f_·(1 − x_wf_) (1)

In order to compare the different samples and drying conditions applied, the moisture represented in the drying curves was calculated relative to the initial moisture (X_w_/X_w0_), in dry basis, for each experiment.

### 2.3. Analytical Determinations

Brassica powders were characterized in terms of physicochemical properties including moisture content, water activity, total soluble solids content, particle size distribution, colour, specific volume and solubility. Technological properties of interaction with water (hydration properties) and oil (emulsifying properties) were also evaluated. Moreover, antioxidant properties and glucosinolate and isothiocyanate contents were assessed.

#### 2.3.1. Physicochemical Properties

Water activity (a_w_) was measured with an Aqualab 4TE dew point hygrometer at 25 °C (Decagon devices Inc., Pullman, Washington, DC, USA). Moisture content (x_w_) was obtained gravimetrically by measuring weight loss before and after drying in a Vaciotem-T vacuum oven (JP Selecta, Barcelona, Spain) (*p* = 10 mmHg) at 60 °C until constant weight, according to the official method of the AOAC 934.06 [30]. Total soluble solids contents (x_ss_) were determined by a thermostatic Abbe refractometer NAR-3T (Atago, Tokyo, Japan) through the measurement of Brix degrees at 20 °C, according to the ISO 1743:1982 method. In dried samples, measurements were obtained from an aqueous extract of soluble solids in a 1:10 (*w/v*) ratio. Particle size distribution was determined by laser diffraction using a Mastersizer 2000 equipment (Malvern Panalytical Ltd., Malvern, UK). The equipment was coupled to a unit Hydro 2000, setting the particle absorption index at 0.1, and the refraction indexes at 1.52 and 1.33 for the sample and the dispersed phase (deionized water), respectively. Particle size results were obtained in terms of equivalent volume mean diameter D[4,3], surface area mean diameter D[3,2] and the distribution percentiles d_10_, d_50_ and d_90_. Colour of samples was measured with a CM-1000R spectrocolorimeter (MINOLTA, Tokyo, Japan) using a D65 illuminant and a 10° angle of vision as reference. To quantify colour, the CIE L*a*b* system was used, where L* is the brightness, a* is the red–green component and b* is the yellow–blue component. Hue (h_ab_) and chrome (C_ab_) attributes were also calculated.

#### 2.3.2. Water Interaction and Oil Emulsifying Properties

Specific volume of powders was assessed by measuring the volume of 5 g of sample in a 10 mL test tube. Solubility was obtained following the method described by Mimouni et al. [31], as the mass fraction of dissolved solids in the rehydrated sample. Hygroscopicity was determined according to the method proposed by Cai and Corke [32], which consisted of measuring the gain of water of certain amount of sample when placed in a hermetic chamber next to a saturated solution of sodium sulphate (Na_2_SO_4_) for one week at room temperature (25 °C). Results were expressed in g of water/100 g of sample. Wettability was obtained from the time in which 2 g of powder became completely wet in a beaker containing 20 mL of distilled water at 25 °C [33]. It refers to the wetting time taken by all powder particles to sink completely, and it is inversely related to wetting time. Swelling capacity (SC) was measured according to Raghavendra et al. [34], as the ratio between the volume of the sample when is immersed in water excess for 18 h at 25 °C and the initial weight of the sample. Results were expressed in mL/g. Water holding capacity (WHC) was calculated as the amount of water retained by the sample without the application of any external force; that is, the ratio between the amount of water contained in 0.2 g of powder hydrated with 10 mL of water for 18 h at 25 °C and the dry weight of the powder after freeze drying [34]. Water retention capacity (WRC) was defined as the amount of water retained by the sample when subjected to an external force such as pressure or centrifugation [34]. Thus, 1 g of powder was added to 10 mL of water allowing hydration for 18 h at 25 °C. After that, the mixture was centrifuged for 30 min at 2000 rpm in a MegafugeTM 16 centrifuge (Thermo Fisher Scientific Inc., Waltham, MA, USA) and the resulting precipitate was weighed and freeze-dried to obtain the dry weight of the sample. WRC was calculated as the ratio between the water retained by the powder and the dry weight of the residue.

Oil holding capacity (OHC) was determined by mixing 0.2 g of powder with 1.5 g of sunflower oil and kept overnight at room temperature. Mixture was centrifuged at 3400 rpm for 5 min, the supernatant was removed and the weight of the precipitate obtained. Results were expressed in g of absorbed oil per g of powder [35]. Emulsifying activity (EA) was measured following the method described by Yasumatsu et al. [36]. A 2% (*w/v*) aqueous powder solution was mixed with 7 mL of sunflower oil and homogenised at 2400 rpm for 5 min in a Reax Top Vortex mixer (HeidolphTM, Schwabach, Germany) and, after that, the mixture was centrifuged at 10,000 rpm for 5 min in a Megafuge^TM^ 16 centrifuge (Thermo Fisher Scientific Inc., Waltham, MA, USA). The volume of the emulsion was measured and referred to the total fluid volume. Emulsifying stability (ES) was measured by the same procedure explained for EA, but in this case the emulsions were heated at 80 °C for 30 min before centrifugation at 2000 rpm for 5 min.

#### 2.3.3. Antioxidant Properties

Antioxidant compounds were extracted by mixing the samples with an 80% (*v/v*) methanol/water solution in a 1:10 (*w/v*) or 2:10 (*w/v*) ratio for powders and raw samples, respectively. The mixture was stirred for 1 h in a horizontal stirrer (Magna Equipments S. L., model ANC10, Barcelona, Spain) and further centrifuged at 10,000 rpm for 5 min in an Eppendorf centrifuge 5804/5804R (Eppendorf SE, Hamburg, Germany).

Total phenolic content was measured using the Folin–Ciocalteu method [36,37]. In total, 0.125 mL of the extract was mixed with 0.5 mL of bidistilled water and 0.125 mL of Folin–Ciocalteu reagent (Scharlab S.L., Barcelona, Spain), and allowed to react for 6 min in darkness. Then, 1.25 mL of 7% (*w/v*) sodium carbonate solution and 1 mL of bidistilled water were added. After 90 min in darkness, absorbance was measured at 760 nm in a Helios Zeta UV/Vis spectrophotometer (Thermo Fisher Scientific Inc., Waltham, MA, USA). Results were expressed in mg of Gallic Acid Equivalents (GAE) per g of dry matter.

Total flavonoid content was measured following the modified method described by Luximon-Ramma et al. [38]. Amounts of 1.5 mL of the extract and 1.5 mL of a 2% (*w/v*) aluminium chloride solution (Thermo Fisher Scientific Inc., Waltham, MA, USA) were mixed and kept in darkness for 10 min. Absorbance was measured at 368 nm in a Helios Zeta UV/Vis spectrophotometer (Thermo Fisher Scientific Inc., Waltham, MA, USA) and results were expressed in mg of Quercetin Equivalents (QE) per g of dry matter.

Antioxidant activity was measured by the DPPH and ABTS radical methods. According to DPPH method [39], 0.1 mL of the extract was mixed with 2.9 mL of a 0.1 mM solution of DPPH (2,2-diphenyl-1-picryl hydrazyl) in methanol (Merck KGaA and affiliates, Darmstadt, Germany). After reaction during 60 min in darkness, the absorbance was measured at 575 nm in a Helios Zeta UV/Vis spectrophotometer (Thermo Fisher Scientific Inc., Waltham, MA, USA). Results were expressed in mg of Trolox Equivalent (TE) per gram of dry matter. For the ABTS method [40], 0.1 mL of the extract was added to 2.9 mL of an ABTS^+^ (VWR International LLC, Radnor, PA, USA) solution in phosphate buffer with an absorbance of 0.70 ± 0.02 at 734 nm. Absorbance was measured in a Helios Zeta UV/Vis sp HeidolphTM, Schwabach spectrophotometer (Thermo Fisher Scientific Inc., Waltham, MA, USA) at 734 nm after 7 min of reaction. Results were expressed in mg of Trolox Equivalent (TE) per g of dry matter.

#### 2.3.4. Glucosinolate and Isothiocyanate Content by High Pressure Liquid Chromatography (HPLC)

Glucosinolate content of powders and fresh (not dehydrated) samples of both broccoli stems and white cabbage outer leaves were analysed by HPLC, following the protocol described by Campas-Baypoli et al. [41] with some modifications.

Sample preparation involved the conversion of glucoraphanin into sulforaphane by means of hydrolysis. In total, 0.5 g of powder or fresh sample was added with 4 mL of acidic water (pH 6). The mixture was incubated in a PRECISTERM water bath (JP Selecta, Barcelona, Spain) at 45 °C for 2 h, to complete hydrolysis. In the present work, additionally, in order to quantify the amount of glucosinolates that were transformed into isothiocyanates during the waste-to-powder transformation process, the isothiocyanate (sulforaphane) content in samples not subjected to the hydrolysis or conversion step was also assessed.

Sulforaphane was extracted by adding 20 mL of dichloromethane to the samples and vortexing for 1 min. After 1 h at room temperature, the solution was filtered using Whatman no. 41 paper. The extract was then purified using 3 mL 7086-03 BAKERBOND^®^ solid-phase extraction (SPE) silica gel (SiOH) disposable columns (JT Baker^®^, Phillipsburg, NJ, USA), previously activated with 3 mL of dichloromethane. The organic extract obtained after filtration was passed through the cartridge, washing the column with 3 mL of ethyl acetate and eluting the sulforaphane with 3 mL of methanol. The sample collected was taken to a Hei-VAP Core rotary evaporator (Heidolph Instruments GmbH & Co., Schwabach, Germany) for evaporation of the organic solvent and subsequently resuspended in 100 μL of acetonitrile. The resulting solution was filtered using 0.45 μm nylon membrane, and 20 μL aliquot of this solution was finally injected onto the column of the HPLC system.

The chromatographic analysis was performed using an Alliance 2995 system with diode array detector (Waters, Milford, MA, USA) and a C18 Luna column (Phenomenex, Torrance, CA, USA). The analyses were carried out under isocratic conditions using as mobile phase a solution of acetonitrile–ultrapure water (30:70, *v/v*) with a flow rate of 0.6 mL/min, and the temperature of the column was set at 36 °C. Sulforaphane was detected at 202 nm and the time between injections was 20 min. All samples were analysed in duplicate.

A calibration line of known concentrations of sulforaphane was prepared using an HPLC standard (Merck KGaA and affiliates, Darmstadt, Germany; >90% purity). Solutions of 1, 3, 5, 7.5, 10, 15, 20, 25, 50 and 75 μg/mL were prepared using acetonitrile as solvent. Chromatogram pick areas were extracted and correlated with the concentrations according to the calibration curve. Sulforaphane recovery percentage was determined by adding a known amount of standard (sulforaphane) to selected samples prior to their SPE. The percentage of recovery was calculated from the ratio between the amount of standard recovered (difference between the amount of standard in the enriched sample and the amount of standard in the non-enriched sample) and the known amount of standard added. The recovery percentage was used to correct the values obtained after identification and quantification of sulforaphane.

### 2.4. Statistical Analysis

All analytical determinations were determined at least in triplicate. Statistical analysis of the results obtained was carried out with Statgraphics Centurion software (Centurion XVII.I version, StatPoint Technologies, Inc., Warrenton, VA, USA). Analyses of variance (one-way ANOVA and multifactorial ANOVA) were performed, having previously checked the normality of the data, and using a confidence level of 95% (*p*-value < 0.05).

## 3. Results and Discussion

### 3.1. Drying and Drying Rate Curves

**Figure 1:**
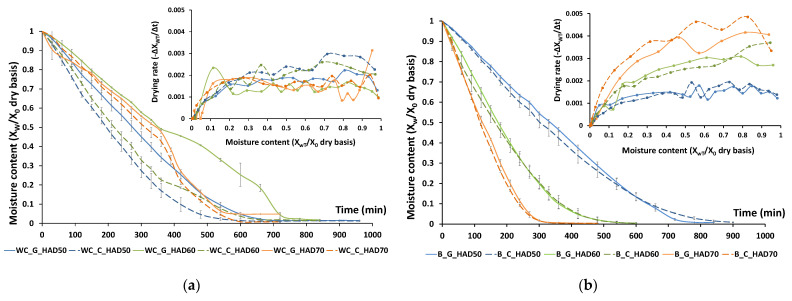
(**a**) Drying curves (moisture content vs. time) and drying rate (drying rate vs. moisture) curves of ground and chopped white cabbage outer leaves (**a**) and broccoli stems (**b**). WC: white cabbage; B: broccoli; G: ground; C: chopped; HAD: hot-air drying at 50, 60 and 70 °C.

Drying curves and drying rate curves obtained at 50, 60 and 70 °C, for a 10 mm thick layer of ground or chopped white cabbage outer leaves or broccoli stems, are shown in Figure 1. Drying curves describe the relationship between the moisture content (expressed on a dry basis) of a sample and the time elapsed, under constant pressure and temperature conditions. Theoretically, hot-air drying of a product takes place in three stages: an initial induction period, followed by the constant drying rate period (CDRP) and a final period of drying at a decreasing rate called the falling drying rate period (FDRP) [42]. However, depending on the characteristics of the sample and the drying conditions, experimental curves may present all or only some of these periods [43].

Drying periods are typically better identified on the drying rate curves. For the brassica residues analysed, the drying rate curves obtained evidenced that most of the water was removed during a CDRP, for most samples. However, some differences were identified between products and drying temperatures. The cabbage residue showed a clearer and longer CDRP, while the broccoli residue did not exhibit this behaviour at higher drying temperatures (60 and 70 °C), and seemed to enter directly into the FDRP. In addition, the drying rates were initially higher for the broccoli residue, thus suggesting that water was more easily removed from broccoli waste than from cabbage waste, especially at higher temperatures, coinciding with the curves that enter directly in the FDRP. This could be due to structural differences between both residual matrices, but also to structural modifications during drying. In fact, a more effective removal of surface water from samples, as observed in the broccoli residue, may cause crusting of the product surface increasing the internal resistance to water transport [44]. Crusting phenomena, also known as case-hardening, imply the accumulation of non-volatile compounds that are carried away by water diffusion, compounds formed because of Maillard reactions or oxidation processes, and structural changes (shrinkage, glass transitions) in the tissue layers close to the surface that hinder the diffusion of water through the matrix [45].

The effect of drying temperature on drying kinetics was evident in all cases, although more pronounced in broccoli, suggesting structural differences among matrices, either initial ones or produced during drying (Figure 1). On the other hand, cabbage showed a more homogeneous behaviour regarding the different drying temperatures applied, which allowed certain influences of the disruption pretreatment to be identified. As observed in Figure 1a, the drying rate was higher when the residue was chopped as compared to ground. This may be due to a more packed matrix in the case of ground cabbage, with the particles closer to each other than in the case of chopped cabbage, which would hinder the diffusion of water to the surface [28]. This effect was less significant in broccoli, where both chopped and ground samples presented quite a similar behaviour. This might be attributed to the tissue structure (stems) being harder and more difficult to disrupt than in cabbage leaves. In a previous study [29] on several vegetables’ residues (leek, cabbage, carrot and celery), chopping allowed drying times to be reduced in most cases, except for carrot, also with a harder structure.

The study of drying kinetics also made it possible to define the time required to reduce initial moisture to reach the target water activity (a_w_ < 0.3) for each residue and drying condition applied. Hence, the time required for white cabbage was 16 h for 50 °C, 14 h for 60 °C and 12 h for 70 °C whereas for broccoli samples, this was 15 h for 50 °C, 10 h for 60 °C and 8 h for 70 °C. Chopped or ground residues required similar drying times at the same drying temperatures.

### 3.2. Physicochemical Characterization

Table 1 shows the results corresponding to water activity (a_w_), moisture content (%) and total soluble solids (x_ss_) of the raw materials (cabbage outer leaves, broccoli stems) and the different powders obtained.

Raw cabbage and broccoli have very high a_w_ values, which indicates perishability and a high risk of spoilage. The drying methods applied allowed an a_w_ in the powders to be reached that guarantees product stability [46]. For a similar drying temperature, and for both cabbage and broccoli powders, those obtained from ground residues showed generally higher moisture values than those obtained from chopped ones. The multifactorial ANOVA analysis confirmed that there were significant differences depending on the milling intensity applied before drying (*p*-value < 0.05). This fact suggests that the structure played a fundamental role in the removal of water. The more packed structure obtained in ground samples could have limited internal water transfer to a higher extent, as compared to chopped ones, in which the presence of interparticle channels might have facilitated the water outflow by diffusion and a capillarity mechanism.

Soluble solid fractions (x_ss_) of powders, obtained from Brix measurements, are also given in Table 1. Powdered foods rich in low molecular weight sugars, such as fruit powders, are very sensitive to environmental conditions so that caking and stickiness may become a problem during storage [47]. Nevertheless, the soluble solid obtained for vegetable powders is relatively low as compared to fruit ones and in line with fruit skins and bagasse [26]. Raw residues showed slightly higher soluble solid contents than powders. This might be due to the structure resulting from the milling and dehydration operation applied, or either to the success of the extraction step from the raw or dried material. No trend was observed depending on drying conditions or pretreatment intensity.

**Figure 2:**
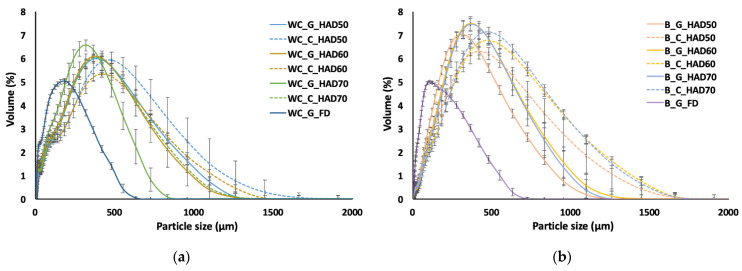
Particle size distribution of powders from white cabbage outer leaves (**a**) and broccoli stems (**b**). The error bars represent the standard deviation of five replicates. WC: white cabbage; B: broccoli; G: ground; C: chopped; HAD: hot-air drying at 50, 60 and 70 °C, FD: freeze drying.

Particle size distributions, measured by the wet procedure, are shown in Figure 2. In this figure, the volume percentage of particles with a specific particle size is plotted. In addition, characteristic parameters D[4,3], D[3,2], d_10_, d_50_ and d_90_ of the powders, are summarized in Table 2, which allowed the identification of statistically significant differences among samples.

The results obtained evidence that both the pretreatment intensity and dehydration technique applied determine powders’ particle size. For both residues, powders obtained by FD presented smaller particle sizes than HAD powders, which could be attributed to the more porous and fragile structure generated during freeze drying, which facilitates further milling [26,48]. Comparing both residues, broccoli powders were usually thicker than cabbage ones. This could be a consequence of faster initial drying rates promoting crusting phenomena, making it more difficult to reach low moisture contents in the inner part of the samples, thus leading to rubbery behaviour and them being more difficult to mill. In contrast, cabbage powders were more homogeneously dried during a longer period, thus obtaining a more brittle material, and being easier to mill. Gulati and Datta [49] investigated case-hardening and texture development during the air drying of food materials and evidenced that, for very low average moisture contents, high drying rates lead to case-hardening phenomena and a product core that remains in the rubbery state, while for lower and more homogeneous drying rates, the entire material transitions into the glassy state. On the other hand, regarding the milling ability, the glassy state is related to a crispier character, whereas in the rubbery state, crispness is lost [50].

The drying temperature also determined particle size, its effect being different depending on the residue. In the case of cabbage leaves, increasing the drying temperature implied a reduction in particle size characteristics; in contrast, broccoli powders presented higher particle sizes when higher temperatures were applied (Table 2). This could be related to the differences observed in the drying behaviour between both residues, as previously explained, since the crusting phenomena occurring in broccoli intensified when increasing temperature. On the other hand, grinding prior to drying resulted in powders with a smaller particle size than chopping, this being more remarkable in broccoli as compared to cabbage. This result is in line with previous studies [29].

**Figure 3:**
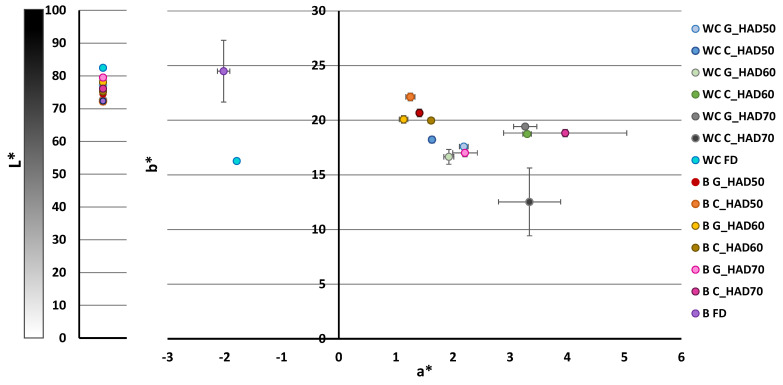
Distribution of L*a*b* coordinates of white cabbage and broccoli waste powders. WC: white cabbage; B: broccoli; G: ground; C: chopped; HAD: hot-air drying at 50, 60 and 70 °C, FD: freeze drying.

Figure 3 show the L*a*b* coordinate distribution of powders, where each point represents a sample. As can be observed, luminosity (L*) presented values in the range of 70–80. In general, luminosity was slightly higher in FD than in HAD powders, as also observed by Xu et al. [51] for hot-air dried at 60 °C (49.5 ± 0.4) and freeze-dried (56.5 ± 0.4) cabbage, and by Vargas et al. [52] for hot-air dried at 70 °C (31 ± 2) and freeze-dried broccoli (47 ± 7). Regarding a* (+redness/−greenness) and b* (+yellowness/−blueness) coordinates, HAD and FD powders were clearly differentiated; however, the drying temperature and intensity of the pretreatment applied did not exhibit a clear trend. a* values were positive for all powders except for the FD ones, indicating a deviation towards the green colour. In the case of the b* coordinate, all values were positive, being generally higher in broccoli powders.

Table 3 shows the results of chroma (C_ab_) and hue (h_ab_) parameters. There were significant differences (*p*-value < 0.05) among samples for both chroma and hue. The higher chroma values found in broccoli powders indicate a higher intensity of saturation. Hue values were generally higher in broccoli. With respect to the drying technique applied, hue values of FD powders were higher than HAD ones, for both vegetable residues.

Table 4 shows the results of the solubility, specific volume, hydration and emulsifying properties of the brassica powders obtained. These are important properties that determine the functionality of powders when used as food ingredients [53].

As expected, FD powders exhibited higher specific volumes than HAD ones due to the air channels generated during sublimation, which produce a more open and porous structure that determines powder volume [29,54]. Regarding solubility, statistically significant differences were not found among powders obtained from the same residue. Solubility is a physical property describing powder behaviour in an aqueous solution, which is related to several characteristics of the powder such as the structure of the polysaccharides [53], powder microstructure or particle size distribution [55,56]. The values obtained in the present work were slightly higher than those reported by Shi et al. [57] for freeze-dried broccoli stem powder (42.1 ± 0.2%).

No statistically significant differences were observed in cabbage powders regarding hygroscopicity (HG), although values slightly increased with temperature in HAD samples. This trend was not observed in broccoli powders. HG was higher in FD powders, mainly in broccoli ones, which could be attributed to the larger surface area of these powders. Similar results were observed by Kapoor and Feng [58] for HAD and FD blueberry and cranberry powders. As reported by Si et al. [55], soluble solids content is also positively related to hygroscopicity.

Wettability did not show a clear trend with respect to the dehydration method used. In contrast, an effect of the pretreatment was identified since chopping led to lower wettability (higher wetting time), as compared to ground. As evidenced by several authors [59,60], wettability is higher for larger particle sizes since large particles imply more interparticle spaces and, therefore, more porosity. This was confirmed in the present work when comparing ground vs. chopped air-dried samples.

Swelling capacity (SC), water retention capacity (WRC) and water holding capacity (WHC) exhibited statistically significant differences among powders; however, no trend was observed with respect to the factors evaluated (dehydration technique or previous milling intensity). Hydration properties play an important role in powder quality since a higher water affinity may improve the functionality of the final product [20,53,61]. According to the literature, intense milling and reduced particle size are negatively related to hydration properties [62]. This phenomenon may be due to the damage of the fiber matrix and the collapse of the pore during grinding. A relationship between particle size and hydration properties was also evidenced by Jongaroontaprangsee et al. [63] on white cabbage outer leaf powders; nevertheless, the same authors did not find a relationship between particle size and hydration properties on citrus powders, concluding that not only particle size but also fiber nature and process history contribute to hydration properties. In fact, it has also been reported that higher drying rates reduce hydration properties [63], a phenomenon that was observed in the present study in the case of broccoli HAD powders obtained at 70 °C. In contrast, and although not significant, FD powders were in the upper range of the values obtained for SC, WHC and WRC. This is in line with other authors who reported that, as compared to FD, HAD reduces the hydration properties and porosity of vegetable and fruit powders [64,65].

In addition to hydration properties, vegetable powders may have the ability to trap fat, which make emulsifying properties interesting parameters for their characterization. Emulsifying properties are affected by the size, shape and superficial area of the particles, as well as by the nature of the fiber present. However, the powders obtained in the present work did not exhibit an interesting behaviour with regard to their emulsifying properties. On the one hand, no results were obtained for the emulsifying activity and stability; on the other hand, small values for oil holding capacity (OHC) were obtained. These values were in the same range as those reported for turnip fibers (between 3.32–6.35 g/g) [66], and slightly lower than those reported for asparagus fibers (between 5.28–8.53 g/g) [67]. Regarding the drying technique applied, FD powders showed better oil absorption capacity than HAD ones, as reported by Que et al. [64].

Indeed, brassica powders exhibited better hydration properties than emulsifying ones. Hydration properties reveal the presence of a greater number of hydrophilic groups and soluble fibers with a high ability to absorb water [20]. On the other hand, both proteins and fibers contribute to emulsion formation due to both their hydrophobic and hydrophilic domains [20,57,65]. The incorporation of ingredients with higher water holding and swelling capacity may improve the viscosity and texture quality [53] of a great variety of foods, especially meat products; hydration properties are also important regarding the consistency of the final product in baking applications [20]. On the other hand, oil absorption properties are important to prevent fat loss during cooking and to enhance food flavour [20,53].

### 3.3. Antioxidant Properties

Total phenols, total flavonoids and the antioxidant capacity (measured by the DPPH and ABTS methods) of the powders and raw materials are shown in Table 5.

Broccoli stems were richer in total phenols and flavonoids than cabbage outer leaves, in agreement with Jaiswal et al. [68]. Results evidenced a different impact of the dehydration technique used on the phenolic and flavonoid content of powders. On the one hand, FD generally maintained the levels of phenolic and flavonoid compounds that were naturally present in the raw sample, suggesting this is a good technique to preserve the original characteristics of the product. However, results were different when the residues were transformed into powders by means of HAD. In general, but more markedly in the case of broccoli stems, drying at 50 and 60 °C reduced the total phenolic content by 4–27%, while drying at 70 °C implied an increase by 23–60%. In fact, the use of high temperatures during drying has been reported to increase the formation of new phenolic substances and contribute to the formation of compounds with high antioxidant activity as a result of Maillard reactions [64,69]. On the other hand, the shorter time needed to complete drying when higher temperatures are used might also have reduced the phenolics’ degradation.

White cabbage powders exhibited a total flavonoid content 1.4 to 2.7 higher than the fresh residue. In the case of broccoli stems, drying at 50 and 60 °C reduced the total flavonoid content by 33–52% and 22–44%, respectively, while drying at 70 °C increased it by 15–76%. Multifactorial ANOVA revealed statistically significant differences for the dehydration factor (*p*-value < 0.05), pointing to HAD70 as the best drying treatment for obtaining powders with the highest content of both total phenols and flavonoids. Regarding disruption pretreatments, chopping generally led to higher phenol and flavonoid content than grinding. This fact could be related to a lower cell tissue damage in the case of chopped samples, so that phenols and flavonoids remain trapped by the structure during drying, thus remaining less susceptible to oxidation during this stage of processing [24].

As for the antioxidant capacity, HAD resulted in powders with a noticeably higher ability to scavenge ABTS free radicals and a slightly higher ability to scavenge DPPH radicals compared to FD. This has previously been reported in similar [29] and other powdered products, from pumpkin [64] or lemon pomace [70], for example. The drying temperatures used in the present study may have promoted the formation of Maillard reaction products, which increases the antioxidant activity [71,72]. As reported by Bernaert et al. [69] processing may result in either a depletion or an increase in antioxidant properties in foods. Thermal treatment can also reduce the action of prooxidant elements, thus contributing to better antioxidant properties. In contrast, the loss of antioxidants due to long processing exposure or the formation of compounds with prooxidant action may lower the antioxidant capacity during processing. Powders from chopped broccoli stems dried with air at 70 °C exhibited the highest antioxidant capacity; in contrast, no statistically significant differences were obtained among temperatures when air drying cabbage leaves. These differences depending on the type of residue could indicate that the antioxidant compounds present in white cabbage were more thermolabile than those present in broccoli stems, or also a reduced generation of new antioxidant compounds during processing. Again, FD kept the antioxidant capacity virtually unchanged, which may be attributed to milder processing conditions, including less exposure to oxygen [51]. Regarding the intensity of tissue disruption prior to drying, chopped samples exhibited higher antioxidant capacity than ground ones. As mentioned before, a higher disruption intensity could imply a loss of antioxidant compounds because of higher tissue de-compartmentation and a larger surface area in contact with drying air, thus boosting oxidative reactions.

### 3.4. Glucosinolate and Isothiocyanate Content

As described in the Materials and Methods section, the protocol published by Campas-Baypoli et al. [41] includes a hydrolysis step for the conversion of glucoraphanin to sulforaphane, which allows the indirect quantification of glucoraphanin. In this work, however, in order to distinguish between the sulforaphane produced by the transformation process of the residue into powder and that resulting from the hydrolysis conversion included in the experimental protocol, sulforaphane content was analysed in the powders and the raw samples both with and without the hydrolysis step. The results of this analysis are presented in Figure S1 as supplementary material, where it is observed that the sulforaphane content of hydrolysed samples (H) was slightly higher, but not significantly, than non-hydrolysed ones (NH) (Figure S1). Differences between H and NH samples were not statistically significant for any of the treatments applied (*p*-value ≥ 0.05), indicating that the hydrolysis stage was not necessary to evaluate the effect of processing or to assess which powder presents better characteristics. Therefore, the values presented in Table 6 correspond to powders, and raw residues, obtained without the hydrolysis step.

The sulforaphane content of broccoli stems was about seven times higher than the values obtained for cabbage outer leaves. Values were in the range of those obtained by Thomas et al. [19] for broccoli stems (591 μg/g_dm_), slightly higher than those reported by Campas-Baypoli et al. [41] (240 μg/g_dm_) and lower than those given by Domínguez-Perles et al. [73] (around 1800 μg/g_dm_) for fresh broccoli stems. Regarding cabbage, the results were in the range of those reported by Lekcharoenkul et al. [74] for fresh and powdered white cabbage outer leaves (60–70 μg/g_dm_). Discrepancies may be due to differences in the extraction and detection methods, as well as differences in the variety of the raw material used or even the sample origin.

In all cases, processing implied a degradation of sulforaphane since the best results were obtained for fresh tissues. On the one hand, drying conditions (temperature, time) could have inhibited the myrosinase action, as described in Vargas et al. [52]. The myrosinase enzyme has an optimal activity at 60 °C but may lose its activity when exposed to higher temperatures or long treatments [74,75]. On the other hand, sulforaphane is a thermosensitive molecule and exposure to temperatures around 60 °C or higher for long periods may adversely affect its stability [8,76]. In this sense, Tanongkankit et al. [75] demonstrated in a previous study the effect of hot-air drying in gradients from 40 to 70 °C on the evolution of sulforaphane, and it was evidenced that sulforaphane is formed at an early stage of drying, but when the temperature increases at the end of the drying process (more than 60 °C), it is degraded. Consequently, only a fraction of sulforaphane remains in the final powder.

Multifactorial ANOVA analysis of the results revealed significant differences (*p*-value < 0.05) regarding the dehydration technique applied. For both residues, sulforaphane was better preserved by FD than HAD. It should be noted that FD is carried out under less aggressive conditions for the vegetal material regarding the temperature and low oxygen conditions, which would result in a greater sulforaphane stability during processing. Among HAD powders, the drying temperature or intensity of previous disruption did not have a statistically significant impact on the amount of sulforaphane contained in the powders. As compared to the raw sample, sulforaphane was better preserved in white cabbage powders (64–73%) than in broccoli ones (60–64%).

## 4. Conclusions

This research made it possible to obtain powdered products from cabbage outer leaves and broccoli stems, and vegetable residues generated during the early stages of processing for fresh and fourth range product commercialization. The results obtained demonstrated the impact of processing on the powders’ properties. Both the dehydration technique and conditions applied, as well as the intensity of the disruption pretreatment, were evidenced to have a significant impact on the relevant properties of powders.

On the one hand, freeze drying was revealed as a good technique to preserve the antioxidant properties of raw products; in contrast, hot-air drying promoted the antioxidant properties due to the generation of new compounds with increased antioxidant activity, especially at high temperatures. The relevance of disruption prior to drying was also demonstrated in properties such as the drying rate, particle size or antioxidant properties. In fact, the interdependence of pretreatment intensity and drying, and its impact on particle size was evidenced, particularly for broccoli stems. In general, chopping facilitates subsequent drying and contributes to a product with a smaller particle size and better antioxidant characteristics than previously ground samples. Other properties, such as water and oil interaction properties mostly depend on the drying technique applied. Regarding potential applications as food ingredients, brassica powders exhibited better hydration than emulsifying properties. Isothiocyanates were identified in all the powders obtained, with broccoli powders being richer than cabbage ones. When transformed into powders, most glucoraphanin was transformed into sulforaphane; nevertheless, processing also had a negative impact on sulforaphane content, mainly attributed to the drying step.

In general terms, it is concluded that white cabbage and broccoli residues produced at the early stages of processing can be successfully transformed into powdered products with interesting properties to be used as functional food ingredients. The reintroduction of these residual organic matter products into the food chain would effectively contribute to the development of more sustainable food systems and is a step towards more sustainable and healthier diets.

## Figures and Tables

**Table 1:** Water activity (a_w_), moisture content (g water/100 g) and soluble solids content (x_ss_ in g soluble solids/g dry matter) of white cabbage (WC) and broccoli (B) samples. G: ground, C: chopped; HAD: hot-air drying at 50, 60 and 70 °C, FD: freeze drying. Mean ± standard deviation of six replicates.

Sample	a_w_	Moisture Content (%)	x_ss_ (g_ss_/g_dm_)
WC_G_HAD50	0.30 ± 0.04 ^bc^	4.3 ± 1.2 ^b^	0.62 ± 0.02 ^ab^
WC_C_HAD50	0.285 ± 0.009 ^abc^	3.2 ± 0.9 ^ab^	0.637 ± 0.004 ^ab^
WC_G_HAD60	0.256 ± 0.014 ^abc^	2.6 ± 1.4 ^ab^	0.652 ± 0.013 ^bc^
WC_C_HAD60	0.254 ± 0.009 ^abc^	2.49 ± 0.12 ^ab^	0.61 ± 0.04 ^ab^
WC_G_HAD70	0.23 ± 0.09 ^a^	3.1 ± 1.1 ^ab^	0.61 ± 0.06 ^ab^
WC_C_HAD70	0.245 ± 0.08 ^ab^	2.6 ± 0.8 ^a^	0.58 ± 0.10 ^a^
WC_G_FD	0.30 ± 0.03 ^c^	3.4 ± 1.4 ^ab^	0.64 ± 0.05 ^bc^
WC (raw)	0.990 ± 0.003 ^d^	91.4 ± 0.2 ^c^	0.70 ± 0.02 ^c^
B_G_HAD50	0.30 ± 0.04 ^ab^	3.7 ± 0.2 ^bc^	0.56 ± 0.03 ^a^
B_C_HAD50	0.312 ± 0.08 ^b^	3.3 ± 0.2 ^ab^	0.60 ± 0.03 ^abc^
B_G_HAD60	0.253 ± 0.007 ^a^	2.3 ± 0.7 ^a^	0.58 ± 0.02 ^ab^
B_C_HAD60	0.283 ± 0.010 ^ab^	2.2 ± 0.64 ^a^	0.59 ± 0.02 ^b^
B_G_HAD70	0.32 ± 0.05 ^ab^	3.8 ± 1.8 ^b^	0.59 ± 0.02 ^b^
B_C_HAD70	0.30 ± 0.04 ^b^	2.6 ± 1.0 ^a^	0.61 ± 0.02 ^b^
B_G_FD	0.298 ± 0.04 ^ab^	2.7 ± 0.4 ^b^	0.608 ± 0.018 ^b^
B (raw)	0.998 ± 0.004 ^c^	92.83 ± 0.09 ^c^	0.70 ± 0.02 ^c^

^a,b,c…^ Different superscript letters in the same column for the same residue indicate statistically significant differences at the 95% confidence level (*p*-value < 0.05).

**Table 2:** Particle size characteristic parameters of white cabbage (WC) and broccoli (B) powders: equivalent volume diameter D[4,3], surface area mean diameter D[3,2], percentiles d_10_, d_50_ and d_90_. HAD: hot-air drying at 50, 60 and 70 °C, FD: freeze drying; C: chopped; G: ground. Mean ± standard deviation.

Sample	D[4,3]	D[3,2]	d10	d50	d90
WC_G_HAD50	306 ± 12 ^c^	48.7 ± 1.1 ^d^	19.2 ± 0.4 ^c^	249 ± 7 ^d^	688 ± 33 ^cd^
WC_C_HAD50	350 ± 49 ^d^	58 ± 4 ^f^	25 ± 2 ^e^	283 ± 38 ^e^	792 ± 117 ^e^
WC_G_HAD60	292 ± 22 ^c^	51.8 ± 1.8 ^e^	20.7 ± 0.4 ^d^	240 ± 14 ^d^	650 ± 59 ^c^
WC_C_HAD60	299 ± 24 ^c^	42.2 ± 0.7 ^b^	16.7 ± 0.2 ^b^	218 ± 13 ^c^	714 ± 66 ^d^
WC_G_HAD70	231 ± 11 ^b^	47.0 ± 1.1 ^cd^	18.7 ± 0.6 ^c^	197 ± 9 ^b^	498 ± 29 ^a^
WC_C_HAD70	294 ± 20 ^c^	45.7 ± 1.6 ^c^	16.9 ± 0.7 ^b^	241 ± 16 ^d^	661 ± 47 ^cd^
WC_G_FD	137 ± 3 ^a^	34.3 ± 0.6 ^a^	13.9 ± 0.2 ^a^	101 ± 2 ^a^	323 ± 8 ^a^
B_G_HAD50	299 ± 13 ^b^	83 ± 4 ^b^	56 ± 3 ^b^	255 ± 8 ^b^	609 ± 36 ^b^
B_C_HAD50	387 ± 37 ^d^	91 ± 5 ^c^	56 ± 3 ^b^	318 ± 27 ^c^	828 ± 94 ^d^
B_G_HAD60	352 ± 18 ^c^	105 ± 2 ^e^	72.2 ± 1.9 ^f^	308 ± 10 ^c^	698 ± 50 ^c^
B_C_HAD60	438 ± 34 ^e^	97 ± 3 ^d^	63 ± 2 ^d^	373 ± 23 ^d^	912 ± 83 ^e^
B_G_HAD70	341 ± 22 ^c^	91 ± 4 ^c^	59 ± 3 ^c^	303 ± 15 ^c^	681 ± 51 ^c^
B_C_HAD70	445 ± 38 ^e^	104 ± 4 ^e^	71 ± 4 ^e^	388 ± 28 ^d^	906 ± 91 ^e^
B_G_FD	145 ± 4 ^a^	42.4 ± 0.4 ^a^	18.3 ± 0.3 ^a^	102 ± 3 ^a^	341 ± 11 ^a^

^a,b,c…^ Different superscript letters in the same column for the same residue indicate statistically significant differences at the 95% confidence level (*p*-value < 0.05).

**Table 3:** Colour parameters of white cabbage (WC) and broccoli (B) powders: chroma (C_ab_) and hue (h_ab_). HAD: hot-air drying at 50, 60 and 70 °C, FD: freeze drying; C: chopped; G: ground. Mean ± standard deviation of three replicates.

Sample	Chroma (C_ab_)	Hue (h_ab_)	Sample	Chroma (C_ab_)	Hue (h_ab_)
WC_G_HAD50	17.7 ± 0.2 ^bc^	82.9 ± 0.2 ^c^	B_G_HAD50	20.7 ± 0.4 ^bc^	86.08 ± 0.05 ^c^
WC_C_HAD50	18.3 ± 0.3 ^bc^	84.88 ± 0.05 ^d^	B_C_HAD50	22.2 ± 0.4 ^c^	86.75 ± 0.16 ^c^
WC_G_HAD60	16.7 ± 0.7 ^b^	83.39 ± 0.06 ^c^	B_G_HAD60	20.1 ± 0.3 ^b^	86.76 ± 0.17 ^c^
WC_C_HAD60	19.02 ± 0.04 ^c^	80.1 ± 0.2 ^b^	B_C_HAD60	20.04 ± 0.02 ^b^	85.37 ± 0.15 ^c^
WC_G_HAD70	19.71 ± 0.16 ^c^	80.5 ± 0.5 ^b^	B_G_HAD70	17.2 ± 0.4 ^a^	82.6 ± 0.6 ^b^
WC_C_HAD70	12 ± 3 ^a^	74.9 ± 1.1 ^a^	B_C_HAD70	19.27 ± 0.16 ^b^	78 ± 3 ^a^
WC_G_FD	16.37 ± 0.09 ^b^	96.26 ± 0.14 ^e^	B_G_FD	24 ± 2 ^d^	94.7 ± 0.4 ^d^

^a,b,c…^ Different superscript letters in the same column for the same residue indicate statistically significant differences at the 95% confidence level (*p*-value < 0.05).

**Table 4:** Results of specific volume, solubility, hydration and water retention properties and emulsifying properties of white cabbage (WC) and broccoli (B) powders. Spec V: specific volume (mL); % solubility expressed as a percentage; HG: hygroscopicity (%) (g_water_/100 g_sample_); Wt: wetting time (min); SC: swelling capacity (mL/g); WHC: water holding capacity (g/g); WRC: water retention capacity (g/g); OHC: oil holding capacity (g/g). G: ground; C: chopped; HAD: hot-air drying at 50, 60 and 70 °C; FD: freeze drying. Mean ± standard deviation of three replicates.

Sample	Spec. V (mL)	Solubility (%)	HG (%)	Wt (min)	SC (mL/g)	WHC (g/g)	WRC (g/g)	OHC (g/g)
WC_G_HAD50	1.80 ± 0.04 ^c^	52 ± 6 ^a^	63.0 ± 0.7 ^a^	8.7 ± 0.9 ^a^	11.17 ± 0.15 ^bc^	18 ± 3 ^ab^	9.0 ± 0.3 ^a^	3.87 ± 0.11 ^b^
WC_C_HAD50	1.807 ± 0.012 ^c^	56 ± 4 ^a^	61.8 ± 0.7 ^a^	9 ± 2 ^a^	11.37 ± 0.06 ^c^	20 ± 7 ^ab^	15 ± 2 ^c^	3.1 ± 0.5 ^a^
WC_G_HAD60	1.51 ± 0.02 ^a^	54 ± 3 ^a^	64.8 ± 0.6 ^ab^	14 ± 6 ^ab^	11.10 ± 0.10 ^b^	15 ± 6 ^a^	10.1 ± 0.7 ^ab^	4.2 ± 0.3 ^b^
WC_C_HAD60	1.53 ± 0.02 ^a^	55 ± 6 ^a^	66.1 ± 1.2 ^ab^	18 ± 4 ^bc^	10.8 ± 0.3 ^a^	20 ± 4 ^ab^	9.9 ± 0.9 ^ab^	3.0 ± 0.4 ^a^
WC_G_HAD70	1.993 ± 0.012 ^d^	55 ± 4 ^a^	68 ± 12 ^ab^	6 ± 2 ^a^	11.10 ± 0.11 ^b^	28 ± 5 ^b^	10 ± 2 ^ab^	4.38 ± 0.10 ^bc^
WC_C_HAD70	1.67 ± 0.03 ^b^	55 ± 3 ^a^	67.1 ± 1.3 ^ab^	29 ± 4 ^c^	11.40 ± 0.10 ^c^	21 ± 6 ^ab^	11.0 ± 1.5 ^ab^	2.9 ± 0.2 ^a^
WC_G_FD	2.96 ± 0.03 ^e^	58 ± 4 ^a^	74 ± 11 ^b^	15 ± 6 ^ab^	10.8 ± 0.2 ^a^	21 ± 7 ^b^	13 ± 3 ^bc^	4.8 ± 0.5 ^c^
B_G_HAD50	1.65 ± 0.04 ^c^	54 ± 4 ^a^	67.9 ± 0.7 ^ab^	2.5 ± 1.5 ^a^	10.70 ± 0.11 ^c^	33 ± 4 ^bc^	13.2 ± 1.6 ^b^	4.85 ± 0.14 ^c^
B_C_HAD50	1.32 ± 0.02 ^a^	45 ± 3 ^a^	70.8 ± 0.6 ^ab^	4 ± 2 ^ab^	11.47 ± 0.06 ^f^	35 ± 10 ^c^	12.5 ± 1.3 ^b^	4.0 ± 0.5 ^b^
B_G_HAD60	1.44 ± 0.04 ^b^	53 ± 11 ^a^	65.6 ± 0.5 ^a^	4.4 ± 1.2 ^ab^	10.93 ± 0.06 ^de^	24 ± 2 ^bc^	11.50 ± 0.14 ^b^	4.2 ± 0.3 ^b^
B_C_HAD60	1.307 ± 0.012 ^a^	56 ± 6 ^a^	65.2 ± 0.6 ^a^	7.79 ± 1.16 ^bc^	10.91 ± 0.12 ^d^	25 ± 4 ^bc^	13 ± 2 ^b^	3.9 ± 0.3 ^b^
B_G_HAD70	1.69 ± 0.06 ^c^	55 ± 4 ^a^	66 ± 3 ^a^	8.9 ± 0.6 ^c^	9.47 ± 0.06 ^b^	20 ± 4 ^ab^	7.7 ± 0.3 ^a^	3.3 ± 0.3 ^a^
B_C_HAD70	1.27 ± 0.03 ^a^	52 ± 7 ^a^	77 ± 14 ^bc^	10 ± 5 ^c^	5.00 ± 0.10 ^a^	12 ± 3 ^a^	6.7 ± 0.5 ^a^	2.81 ± 0.15 ^a^
B_G_FD	3.98 ± 0.02 ^d^	54 ± 6 ^a^	84.3 ± 0.7 ^c^	1.9 ± 0.9 ^a^	11.03 ± 0.06 ^e^	32 ± 4 ^bc^	12.7 ± 0.7 ^b^	6.5 ± 0.3 ^d^

^a,b,c…^ Different superscript letters in the same column for the same residue indicate statistically significant differences at the 95% confidence level (*p*-value < 0.05).

**Table 5:** Results of antioxidant properties of the raw and powdered residues of white cabbage (WC) and broccoli (B). HAD: hot-air drying at 50, 60 and 70 °C, FD: freeze drying; C: chopped; G: ground. Mean ± standard deviation.

Sample	Total Phenols (mg GAE/g_dm_)	Total Flavonoids (mg QE/g_dm_)	DPPH (mg TE/g_dm_)	ABTS (mg TE/g_dm_)
WC_G_HAD50	3.3 ± 0.2 ^a^	2.5 ± 0.7 ^a^	1.5 ± 0.3 ^ab^	49 ± 3 ^b^
WC_C_HAD50	3.7 ± 0.8 ^a^	4.0 ± 1.0 ^cd^	3.0 ± 0.6 ^d^	51 ± 10 ^b^
WC_G_HAD60	3.3 ± 0.5 ^a^	2.6 ± 0.7 ^ab^	1.3 ± 0.3 ^a^	49 ± 6 ^b^
WC_C_HAD60	4.1 ± 1.1 ^ab^	3.7 ± 0.6 ^bc^	2.17 ± 0.14 ^c^	59.6 ± 1.2 ^b^
WC_G_HAD70	4.8 ± 0.9 ^bc^	4.1 ± 1.2 ^cd^	1.4 ± 0.7 ^a^	48 ± 6 ^b^
WC_C_HAD70	5.4 ± 0.4 ^c^	4.9 ± 0.6 ^d^	2.048 ± 0.016 ^abc^	58 ± 15 ^b^
WC_G_FD	4.0 ± 1.2 ^ab^	1.8 ± 0.4 ^a^	1.85 ± 0.14 ^abc^	25 ± 6 ^a^
WC raw	3.9 ± 0.4 ^a^	1.8 ± 0.3 ^a^	2.08 ± 0.17 ^bc^	30 ± 2 ^a^
B_G_HAD50	3.5 ± 0.6 ^a^	2.2 ± 0.4 ^a^	2.60 ± 1.05 ^ab^	69 ± 4 ^b^
B_C_HAD50	4.8 ± 0.5 ^b^	3.1 ± 0.2 ^b^	3.1 ± 0.4 ^bcd^	83 ± 11 ^c^
B_G_HAD60	3.9 ± 0.6 ^ab^	2.6 ± 0.4 ^ab^	2.1 ± 0.3 ^a^	88 ± 11 ^cd^
B_C_HAD60	4.4 ± 0.5 ^ab^	3.6 ± 0.8 ^c^	2.7 ± 0.5 ^abc^	98 ± 14 ^de^
B_G_HAD70	6.7 ± 1.2 ^c^	5.3 ± 0.4 ^e^	2.4 ± 0.4 ^ab^	90 ± 10 ^cd^
B_C_HAD70	8 ± 2 ^d^	8.1 ± 0.4 ^f^	3.40 ± 0.17 ^cd^	108 ± 8 ^e^
B_G_FD	4.3 ± 1.2 ^ab^	5.50 ± 0.03 ^e^	2.9 ± 0.9 ^bcd^	50 ± 5 ^a^
B raw	5.0 ± 1.0 ^b^	4.6 ± 0.7 ^d^	3.7 ± 0.3 ^d^	34.0 ± 1.6 ^a^

^a,b,c…^ Different superscript letters in the same column for the same residue indicate statistically significant differences at the 95% confidence level (*p*-value < 0.05).

**Table 6:** Sulforaphane content (µg/g_dm_) of powders and raw samples. WC: white cabbage, B: broccoli; G: ground, C: chopped; HAD: hot-air drying at 50, 60 and 70 °C, FD: freeze drying. Mean ± standard deviation.

Sample	White Cabbage (WC)	Broccoli (B)
G_HAD50	68.7 ± 0.5 ^ab^	470.2 ± 1.1 ^a^
C_HAD50	67 ± 9 ^a^	479 ± 3 ^a^
G_HAD60	71 ± 6 ^ab^	476 ± 10 ^a^
C_HAD60	70 ± 6 ^ab^	472 ± 16 ^a^
G_HAD70	69 ± 8 ^ab^	493 ± 52 ^a^
C_HAD70	73 ± 3 ^ab^	461 ± 9 ^a^
FD	77 ± 4 ^ab^	506 ± 11 ^a^
Raw	105 ± 1 ^c^	787 ± 5 ^b^

^a,b,c^ Different superscript letters for a similar residue indicate statistically significant differences at the 95% confidence level (*p*-value < 0.05).

## Data Availability

Data available from the authors, upon reasonable request.

## Supplementary Materials

The following supporting information can be downloaded at: https://www.mdpi.com/article/10.3390/foods11223663/s1, Figure S1: Sulforaphane content (μg/g_dm_) in cabbage (a) and broccoli (b) samples. H: with hydrolysis; NH: without hydrolysis. WC: white cabbage; B: broccoli; G: ground; C: chopped; HAD: hot-air drying; FD: freeze drying.

## References

[1] Burel C., Boujar T., Escaffre A.M., Kaushik S.J., Boeuf G., Mol K.A., Van der Geyten S., Kühn E.R. (2000). Dietary low-glucosinolate rapeseed meal affects thyroid status and nutrient utilization in rainbow trout (*Oncorhynchus mykiss*). Br. J. Nutr..

[2] Ahuja I., Rohloff J., Bones M. (2010). Defence mechanisms of Brassicaceae: Implications for plant-insect interactions and potential for integrated pest management. A review. Agron. Sustain. Dev..

[3] Bohinc T., Ban S.G., Ban D., Trdan S. (2012). Glucosinolates in plant protection strategies: A review. Arch. Biol. Sci..

[4] Ishida M., Hara M., Fukino N., Kakizaki T., Morimitsu Y. (2014). Glucosinolate metabolism, functionality and breeding for the improvement of Brassicaceae vegetables. Breed Sci..

[5] Prieto M.A., López C.J., Simal-Gandara J. (2019). Glucosinolates: Molecular structure, breakdown, genetic, bioavailability, properties and healthy and adverse effects. Adv. Food Nutr. Res..

[6] Bischoff K.L. (2016). Glucosinolates. Nutraceuticals Effic. Saf. Toxic..

[7] Zukalová H., Vašák J. (2002). The role and effects of glucosinolates of Brassica species—A review. Plant Soil Environ..

[8] Kuljarachanan T., Chiewchan N., Devahastin S. (2019). Profiles of major glucosinolates in different parts of white cabbage and their evolutions during processing into vegetable powder by various methods. Int. Food Res. J..

[9] Textor S., Gershenzon J. (2009). Herbivore induction of the glucosinolate-myrosinase defense system: Major trends, biochemical bases and ecological significance. Phytochem. Rev..

[10] Angelino D., Jeffery E. (2014). Glucosinolate hydrolysis and bioavailability of resulting isothiocyanates: Focus on glucoraphanin. J. Funct. Foods.

[11] Wagner A.E., Terschluesen A.M., Rimbach G. (2013). Health promoting effects of brassica-derived phytochemicals: From chemopreventive and anti-inflammatory activities to epigenetic regulation. Oxid. Med. Cell. Longev..

[12] Halkier B.A., Gershenzon J. (2006). Biology and biochemistry of glucosinolates. Annu. Rev. Plant Biol..

[13] Gupta P., Kim B., Kim S.H., Srivastava S.K. (2014). Molecular targets of isothiocyanates in cancer: Recent advances. Mol. Nutr. Food Res..

[14] Joensuu K., Hartikainen H., Karppinen S., Jaakkonen A.K., Kuoppa-aho M. (2021). Developing the collection of statistical food waste data on the primary production of fruit and vegetables. Environ. Sci. Pollut. Res..

[15] FAOSTAT. http://www.fao.org/faostat/en/#data/QC/visualize.

[16] FAO and the Sustainable Development Goals|FAO|Food and Agriculture Organization of the United Nations. https://www.fao.org/about/strategy-programme-budget/strategic-framework/fao-sdg/en/.

[17] Ferreira M.S.L., Santos M.C.P., Moro T.M.A., Basto G.J., Andrade R.M.S., Gonçalves E.C.B.A. (2015). Formulation and characterization of functional foods based on fruit and vegetable residue flour. J. Food Sci. Technol..

[18] Neacsu M., Vaughan N., Raikos V., Multari S., Duncan G.J., Duthie G.G., Russell W.R. (2015). Phytochemical profile of commercially available food plant powders: Their potential role in healthier food reformulations. Food Chem..

[19] Thomas M., Badr A., Desjardins Y., Gosselin A., Angers P. (2018). Characterization of industrial broccoli discards (*Brassica oleracea* var. italica) for their glucosinolate, polyphenol and flavonoid contents using UPLC MS/MS and spectrophotometric methods. Food Chem..

[20] Mokhtar S.M., Swailam H.M., Embaby H.E.S. (2018). Physicochemical properties, nutritional value and techno-functional properties of goldenberry (*Physalis peruviana*) waste powder concise title: Composition of goldenberry juice waste. Food Chem..

[21] Karam M.C., Petit J., Zimmer D., Baudelaire Djantou E., Scher J. (2016). Effects of drying and grinding in production of fruit and vegetable powders: A review. J. Food Eng..

[22] Yu J., Vallad G.E., Boyd N.S. (2019). Evaluation of allyl isothiocyanate as a soil fumigant for tomato (*Lycopersicon esculentum* Mill.) production. Plant Dis..

[23] Liu Y., Chen W., Fan L. (2022). Effects of different drying methods on the storage stability of barley grass powder. J. Sci. Food Agric..

[24] Dovene A.K., Wang L., Bokhary S.U.F., Madebo M.P., Zheng Y., Jin P. (2019). Effect of Cutting Styles on Quality and Antioxidant Activity of Stored Fresh-Cut Sweet Potato (*Ipomoea batatas* L.) Cultivars. Foods.

[25] Ramos I.N., Brandão T.R.S., Silva C.L.M. (2003). Structural Changes During Air Drying of Fruits and Vegetables. Food Sci. Technol. Int..

[26] Bas-Bellver C., Andrés C., Seguí L., Barrera C., Jiménez-Hernández N., Artacho A., Betoret N., Gosalbes M.J. (2020). Valorization of Persimmon and Blueberry Byproducts to Obtain Functional Powders: In Vitro Digestion and Fermentation by Gut Microbiota. J. Agric. Food Chem..

[27] Seguí L., Bas-Bellver C., Barrera C., Betoret N., Ramadan M.F., Farag M.A. (2022). Valorization of Persimmon (Diospyros kaki) Wastes to Be Used as Functional Ingredients. Mediterranean Fruits Bio-Wastes.

[28] Ramírez-Pulido B., Bas-Bellver C., Betoret N., Barrera C., Seguí L. (2021). Valorization of Vegetable Fresh-Processing Residues as Functional Powdered Ingredients. A Review on the Potential Impact of Pretreatments and Drying Methods on Bioactive Compounds and Their Bioaccessibility. Front. Sustain. Food Syst..

[29] Bas-Bellver C., Barrera C., Betoret N., Seguí L. (2020). Turning Agri-Food Cooperative Vegetable Residues into Functional Powdered Ingredients for the Food Industry. Sustainability.

[30] Association of Official Analytical Chemist Official Methods of Analysis (2000). AOAC Official Method 934.06, Moisture in Dried Fruits.

[31] Mimouni A., Deeth H.C., Whittaker A.K., Gidley M.J., Bhandari B.R. (2009). Rehydration process of milk protein concentrate powder monitored by static light scattering. Food Hydrocoll..

[32] Cai Y.Z., Corke H. (2000). Production and Properties of Spray-dried Amaranthus Betacyanin Pigments. J. Food Sci..

[33] Freudig B., Hogekamp S., Schubert H. (1999). Dispersion of powders in liquids in a stirred vessel. Chem. Eng. Process..

[34] Raghavendra S.N., Ramachandra Swamy S.R., Rastogi N.K., Raghavarao K.S., Kumar S., Tharanathan R.N. (2006). Grinding characteristics and hydration properties of coconut residue: A source of dietary fiber. J. Food Eng..

[35] Garau M.C., Simal S., Rosselló C., Femenia A. (2007). Effect of air-drying temperature on physico-chemical properties of dietary fibre and antioxidant capacity of orange (*Citrus aurantium* v. Canoneta) by-products. Food Chem..

[36] Yasumatsu K., Sawada K., Moritaka S., Misaki M., Toda J., Wada T., Ishii K. (1972). Whipping and Emulsifying Properties of Soybean Products. Food Nutr. (Roma).

[37] Singleton V.L., Orthofer R., Lamuela-Raventós R.M. (1999). Analysis of total phenols and other oxidation substrates and antioxidants by means of folin-ciocalteu reagent. Methods Enzymol..

[38] Luximon-Ramma A., Bahorun T., Soobrattee M.A., Aruoma O.I. (2002). Antioxidant Activities of Phenolic, Proanthocyanidin, and Flavonoid Components in Extracts of Cassia fistula. J. Agric. Food Chem..

[39] Brand-Williams W., Cuvelier M.E., Berset C. (1995). Use of a free radical method to evaluate antioxidant activity. LWT-Food Sci. Technol..

[40] Re R., Pellegrini N., Proteggente A., Pannala A., Yang M., Rice-Evans C. (1999). Antioxidant activity applying an improved ABTS radical cation decolorization assay. Free Radic. Biol. Med..

[41] Campas-Baypoli O.N., Sánchez-Machado D.I., Bueno-Solano C., Ramírez-Wong B., López-Cervantes J. (2010). HPLC method validation for measurement of sulforaphane level in broccoli by-products. Biomed. Chromatogr..

[42] Doran M.P. (2013). Bioprocess Engineering Principles.

[43] Inyang U.E., Oboh I.O., Etuk B.R., Inyang U.E., Oboh I.O., Etuk B.R. (2018). Kinetic Models for Drying Techniques—Food Materials. Adv. Chem. Eng. Sci..

[44] Maskan M. (2001). Drying, shrinkage and rehydration characteristics of kiwifruits during hot air and microwave drying. J. Food Eng..

[45] Tello-Ireland C., Lemus-Mondaca R., Vega-Gálvez A., López J., di Scala K. (2011). Influence of hot-air temperature on drying kinetics, functional properties, colour, phycobiliproteins, antioxidant capacity, texture and agar yield of alga Gracilaria chilensis. LWT-Food Sci. Technol..

[46] Santos P.H.S., Silva M.A. (2008). Retention of Vitamin C in Drying Processes of Fruits and Vegetables—A Review. Dry. Technol..

[47] Mosquera L.H., Moraga G., Martínez-Navarrete N. (2012). Critical water activity and critical water content of freeze-dried strawberry powder as affected by maltodextrin and arabic gum. Food Res. Int..

[48] van Buggenhout S., Lille M., Messagie I., von Loey A., Autio K., Hendrickx M. (2006). Impact of pretreatment and freezing conditions on the microstructure of frozen carrots: Quantification and relation to texture loss. Eur. Food Res. Technol..

[49] Gulati T., Datta A.K. (2015). Mechanistic understanding of case-hardening and texture development during drying of food materials. J. Food Eng..

[50] Salvador A., Camacho M.M., Martínez-Navarrete N. (2022). Influence of formulation on the quality and stability of a freeze-dried Mandarin product. Curr. Res. Food Sci..

[51] Xu Y., Xiao Y., Lagnika C., Li D., Liu C., Jiang N., Song J., Zhang M. (2020). A comparative evaluation of nutritional properties, antioxidant capacity and physical characteristics of cabbage (*Brassica oleracea* var. Capitate var L.) subjected to different drying methods. Food Chem..

[52] Vargas L., Kapoor R., Nemzer B., Feng H. (2022). Application of different drying methods for evaluation of phytochemical content and physical properties of broccoli, kale, and spinach. LWT.

[53] de Moraes Crizel T., Hermes V.S., de Oliveira Rios A., Flôres S.H. (2016). Evaluation of bioactive compounds, chemical and technological properties of fruits byproducts powder. J. Food Sci. Technol..

[54] Krokida M.K., Karathanos V.T., Maroulis Z.B. (1998). Effect of freeze-drying conditions on shrinkage and porosity of dehydrated agricultural products. J. Food Eng..

[55] Si X., Chen Q., Bi J., Wu X., Yi J., Zhou L., Li Z. (2006). Comparison of different drying methods on the physical properties, bioactive compounds and antioxidant activity of raspberry powders. J. Sci. Food Agric..

[56] Calabuig-Jiménez L., Indira Hinestroza-Córdoba L., Barrera C., Seguí L., Betoret N. (2022). Effects of Processing and Storage Conditions on Functional Properties of Powdered Blueberry Pomace. Sustainability.

[57] Shi M., Hlaing M.M., Ying D.Y., Ye J.H., Sanguansri L., Augustin M.A. (2019). New food ingredients from broccoli by-products: Physical, chemical and technological properties. Int. J. Food Sci. Technol..

[58] Kapoor R., Feng H. (2022). Characterization of physicochemical, packing and microstructural properties of beet, blueberry, carrot and cranberry powders: The effect of drying methods. Powder Technol..

[59] Forny L., Marabi A., Palzer S. (2011). Wetting, disintegration and dissolution of agglomerated water soluble powders. Powder Technol..

[60] Camacho M.M., Silva-Espinoza M.A., Martínez-Navarrete N. (2022). Flowability, Rehydration Behaviour and bioactive Compounds of an Orange Powder Product as Affected by Particle Size. Food Bioprocess. Technol..

[61] Olukomaiya O.O., Adiamo O.Q., Fernando W.C., Mereddy R., Li X., Sultanbawa Y. (2020). Effect of solid-state fermentation on proximate composition, anti-nutritional factor, microbiological and functional properties of lupin flour. Food Chem..

[62] Kethireddipalli P., Hung Y.C., Phillips R.D., McWatters K.H. (2002). Evaluating the Role of Cell Wall Material and Soluble Protein in the Functionality of Cowpea (*Vigna unguiculata*) Pastes. J. Food Sci..

[63] Jongaroontaprangsee S., Tritrong W., Chokanaporn W., Methacanon P., Devahastin S., Chiewchan N. (2007). Effects of Drying Temperature and Particle Size on Hydration Properties of Dietary Fiber Powder from Lime and Cabbage By-Products. Int. J. Food Prop..

[64] Que F., Mao L., Fang X., Wu T. (2008). Comparison of hot air-drying and freeze-drying on the physicochemical properties and antioxidant activities of pumpkin (*Cucurbita moschata* Duch.) flours. Int. J. Food Sci. Technol..

[65] Hinestroza-Córdoba L.I., Serna S.D., Seguí L., Barrera C., Betoret N. (2020). Characterization of Powdered Lulo (*Solanum quitoense*) Bagasse as a Functional Food Ingredient. Foods.

[66] Ma R., Chen J., Zhou X., Lin H., Gao Q., Peng X., Tanokura M., Xue Y. (2021). Effect of chemical and enzymatic modifications on the structural and physicochemical properties of dietary fiber from purple turnip (*Brassica rapa* L.). LWT.

[67] Fuentes-Alventosa J.M., Rodríguez-Gutiérrez G., Jaramillo-Carmona S., Espejo-Calvo J.A., Rodríguez-Arcos R., Fernández-Bolaños J., Guillén-Bejarano R., Jiménez-Araujo A. (2009). Effect of extraction method on chemical composition and functional characteristics of high dietary fibre powders obtained from asparagus by-products. Food Chem..

[68] Jaiswal A.K., Abu-Ghannam N., Gupta S. (2012). A comparative study on the polyphenolic content, antibacterial activity and antioxidant capacity of different solvent extracts of Brassica oleracea vegetables. Int. J. Food Sci. Technol..

[69] Bernaert N., de Clercq H., van Bockstaele E., de Loose M., van Droogenbroeck B. (2013). Antioxidant changes during postharvest processing and storage of leek (*Allium ampeloprasum* var. *porrum*). Postharvest Biol. Technol..

[70] Papoutsis K., Pristijono P., Golding J.B., Stathopoulos C.E., Bowyer M.C., Scarlett C.J., Vuong Q.V. (2017). Effect of vacuum-drying, hot air-drying and freeze-drying on polyphenols and antioxidant capacity of lemon (*Citrus limon*) pomace aqueous extracts. Int. J. Food Sci. Technol..

[71] Gahler S., Otto K., Böhm V. (2003). Alterations of vitamin C, total phenolics, and antioxidant capacity as affected by processing tomatoes to different products. J. Agric. Food Chem..

[72] Chen M.L., Yang D.J., Liu S.C. (2011). Effects of drying temperature on the flavonoid, phenolic acid and antioxidative capacities of the methanol extract of citrus fruit (*Citrus sinensis* (L.) Osbeck) peels. Int. J. Food Sci. Technol..

[73] Domínguez-Perles R., Martínez-Ballesta M.C., Carvajal M., García-Viguera C., Moreno D.A. (2010). Broccoli-derived by-products—A promising source of bioactive ingredients. J. Food Sci..

[74] Lekcharoenkul P., Tanongkankit Y., Chiewchan N., Devahastin S. (2014). Enhancement of sulforaphane content in cabbage outer leaves using hybrid drying technique and stepwise change of drying temperature. J. Food Eng..

[75] Tanongkankit Y., Chiewchan N., Devahastin S. (2011). Evolution of anticarcinogenic substance in dietary fibre powder from cabbage outer leaves during drying. Food Chem..

[76] van Eylen D., Oey I., Hendrickx M., van Loey A. (2007). Kinetics of the stability of broccoli (*Brassica oleracea* Cv. Italica) myrosinase and isothiocyanates in broccoli juice during pressure/temperature treatments. J. Agric. Food Chem..

