# Supplementary material for: Impact of Disruption and Drying Conditions on Physicochemical, Functional and Antioxidant Properties of Powdered Ingredients Obtained from Brassica Vegetable By-Products
Source: Foods. 2022 Nov 16;11(22):3663. doi: 10.3390/foods11223663 (PMC9689784; doi:10.3390/foods11223663)
Supplement: Supplementary file 1 [file foods-11-03663-s001.zip › foods-2014326-supplementary.pdf]

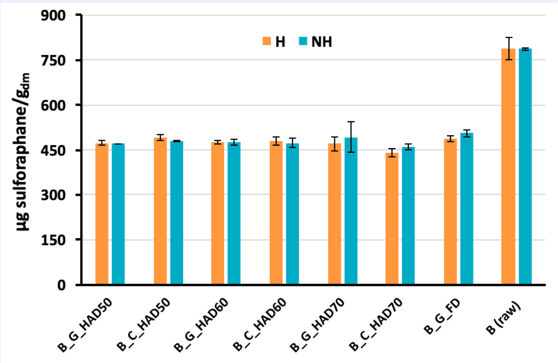

(**a**)

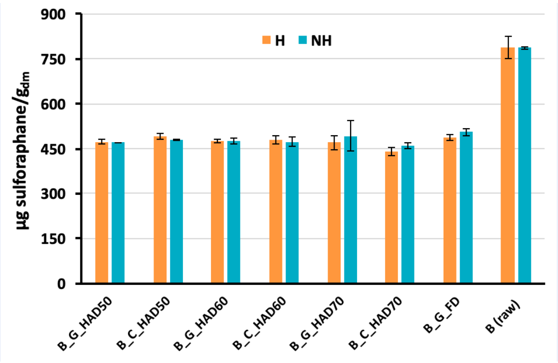

(**b**)

Figure S1. Sulforaphane content (µg/gdm) in cabbage (a) and broccoli (b) samples. H: with hydrolysis; NH: without hydrolysis. WC: white cabbage; B: broccoli; G: ground; C: chopped; HAD: hot-air drying; FD: freeze drying.
